# ICD‐11 ‘mixed depressive and anxiety disorder’ is clinical rather than sub‐clinical and more common than anxiety and depression in the general population

**DOI:** 10.1111/bjc.12321

**Published:** 2021-07-17

**Authors:** Mark Shevlin, Philip Hyland, Emma Nolan, Marcin Owczarek, Menachem Ben‐Ezra, Thanos Karatzias

**Affiliations:** ^1^ Ulster University Coleraine UK; ^2^ Maynooth University Ireland; ^3^ Ariel University Israel; ^4^ Napier University UK

**Keywords:** ICD‐11 mixed depressive and anxiety disorder, sub‐clinical, factor mixture models

## Abstract

**Background:**

The new International Classification of Diseases was published in 2018 (ICD‐11; World Health Organization, 2018) and now includes ‘Mixed depressive and anxiety disorder’ (6A73: MDAD) designated as a mood disorder. This disorder is defined by symptoms of both anxiety and depression occurring more days than not, for a period of two weeks, and neither set of symptoms considered separately reaches a diagnostic threshold for either disorder. However, to date no study has examined the validity of these guidelines in a general population sample.

**Methods:**

Using Goldberg et al.’s (2017) guidelines regarding measurement of depression and anxiety, this study used factor mixture modelling (FMM) to examine the validity of the ICD‐11 criteria of MDAD. Symptom endorsement rates are provided as well as demographic predictors and somatization outcomes.

**Results:**

Fit indices suggested the two‐factor four‐class solution was the best balance between model complexity and model fit. The results did not support a class that is subsyndromal to both anxiety and depression. On the contrary, we suggest that there exists a ‘Comorbid’ class that represents endorsement of both anxiety and depression symptoms at a higher level when compared to both ‘anxiety’ and ‘depression’ groups. Demographic predictors, as well as somatization and functional impairment outcomes, provided support for this FMM solution.

**Conclusions:**

The ‘Comorbid’ group was the largest symptomatic group and had the highest levels of both anxiety and depression symptoms. Importantly, this group was larger than either the ‘anxiety’ or ‘depression’ group and was associated with high levels of functional impairment and somatization.

## Background

In 2018, the 11th version of the International Classification of Diseases was published (World Health Organization, 2018). Section 06, ‘Mental, Behavioural or Neurodevelopmental Disorders’, includes descriptions of ‘Single episode depressive disorder’ (6A70) and ‘Generalised anxiety disorder’ (6B00) under the Mood Disorders and Anxiety or Fear‐Related Disorders, respectively. There is also a ‘Mixed depressive and anxiety disorder’ (6A73: MDAD) designated as a mood disorder, similar to the ‘Mixed anxiety and depressive disorder’ (F41.2) that was under the ‘Neurotic, Stress‐Related, and Somatoform Disorders’ section in ICD‐10. MDAD is described as being ‘…characterized by symptoms of both anxiety and depression more days than not for a period of two weeks or more. Neither set of symptoms, considered separately, is sufficiently severe, numerous, or persistent to justify a diagnosis of a depressive episode, dysthymia, or an anxiety and fear‐related disorder. Depressed mood or diminished interest in activities must be present accompanied by additional depressive symptoms as well as multiple symptoms of anxiety’. There must also be evidence of functional impairment and no indications of bipolar disorder.

The MDAD disorder reflects the high level of comorbidity between depression and anxiety at both the diagnostic and symptom level (Jacobson & Newman, 2017; Möller et al., 2016), and that sub‐clinical manifestations of the disorders are clinically important. For example, sub‐clinical anxiety and depression have been shown to be a major cause of psychiatric morbidity in primary care (Das‐Munshi et al., 2008) and comorbid sub‐threshold anxiety and depression is associated with treatment‐seeking and work impairment (Preisig, Merikangas, & Angst, 2001). The clinical relevance of acknowledging co‐occurring anxiety and depression was evidenced by Lam et al. (2013) who reported ‘overwhelming support’ (p. 76) from an international sample of primary care professionals on the draft ICD‐11 proposal to include ‘anxious depression’ as a disorder. Furthermore, a global survey of psychiatrists from 44 countries reported that mixed anxiety and depressive disorder (MDAD) was one of the most commonly used diagnosis (Reed, Mendonça Correia, Esparza, Saxena, & Maj, 2011).

Möller et al. (2016) reviewed MDAD as a diagnostic category in terms of its history, clinical relevance, nosology, and treatment. However, there has only been a small number of studies that have aimed to test whether there is an identifiable group in community samples that have a symptom profile consistent with MDAD, as now described by the ICD‐11. Hettema, Aggen, Kubarych, Neale, and Kendler (2015) implemented a restricted latent class analysis (LCA), allowing for the specification of classes of individuals that had a prior diagnosis, using 14 symptoms of major depression, and 18 symptoms of generalized anxiety disorder based on DSM‐III‐R (American Psychiatric Association, 1987). Analyses were based on a sample of 8952 twin pairs from the Virginia Adult Twin Study of Psychiatric and Substance‐Use Disorders (Prescott & Kendler, 1999). A class representing MDAD was found and the prevalence was 11%. Belonging to this class was associated with being female, being of younger age, and possessing a history of adult trauma. Das‐Munshi et al., (2008) used categorization rules and LCA based on 14 ICD–10 diagnoses of common mental health disorders generated from the Clinical Interview Schedule‐Revised (CIS–R: Lewis, Pelosi, Araya, & Dunn, 1992), using data from the National Psychiatric Morbidity survey (*N* = 8580: Jenkins et al., 1997). They identified a group of participants (8.8%) who were labelled as ‘Mixed anxiety and depression’ (MAD) on the basis of an overall CIS‐R score greater than 12 (indicting clinically relevant psychiatric morbidity) but failing to meet ICD–10 diagnostic criteria for any mental health disorder. An LCA with five classes was also reported, and the participants in the MAD group were roughly equally distributed across a ‘Distress’ class (high anxiety, worry, depressive ideas, irritability, sleep, and concentration problems) and a large ‘Non‐cases’ class (low levels of anxiety and depression). This suggests that the categorisation based on the CIS–R cut‐off scores does not reflect naturally occurring groups revealed in the LCA.

It could be argued that the categorization method used by Das‐Munshi et al., (2008) to identify MAD cases precluded the identification of co‐occurring anxiety and depression at a clinical level. Goldberg et al. (2017), in a study based on 1488 primary care patients in four middle‐income countries who were suspected of experiencing ‘psychological distress’ (p. 202), did not apply such categorization rules when examining levels of anxiety and depression based on the CIS‐R. It was found that co‐occurring clinically relevant anxiety and depression was more common (48.7%) than either only anxiety (20.0%) or depression (7.8%). It was concluded that ‘…anxiety and depression are by no means distinct’ (p. 204). So, when anxiety and depression are examined without a priori categorization rules being applied, it appears that clinical level mixed anxiety‐depression is more common than the ‘pure’ forms of the disorders.

To date there has been no general population‐level analysis of ICD‐11 anxiety and depression symptoms. Therefore, this study employed factor mixture modelling (FMM) to determine if a class representing MDAD could be identified using Goldberg’s (2017) newly developed ICD‐11‐based anxiety and depression scale within a nationally representative sample of Irish adults. FMM allows the underlying structure of a set of symptoms to be represented simultaneously as categorical and dimensional (Clark, 2010). The categorical aspect of the model allows for classification of groups of individuals into latent classes, and the continuous component allows for the variation in severity at the symptom/item level to be modelled as dimensional latent variables (Clarke, 2010). This approach is more appropriate than latent class analysis at it does not require the dichotomization of continuous or ordinal indicators and does not require the excessively restrictive assumption of within‐class independence that latent profile analysis requires (Macia & Wickham, 2019).

Based on the diagnostic rules of the ICD‐11, it was hypothesized that five classes would be identified including (1) a ‘non‐symptomatic’ class reporting low symptom severity for anxiety and depression, (2) an ‘anxiety class’ with relatively higher severity levels of anxiety than depression, (3) a ‘depression class’ with relatively higher severity levels of depression than anxiety, (4) a ‘comorbid class’ with high levels of anxiety than depression, and (5) a ‘MDAD class’ who would be characterized by having higher levels of symptom severity of anxiety and depression than those who are non‐symptomatic, but lower than those who have a profile of symptom severity consistent with clinically relevant anxiety and/or depression.

The second aim was to assess the degree to which demographic and stress/trauma‐related variables were associated with latent class membership. Based on available evidence, it was hypothesized that female sex (Albert, 2015), younger age (Jorm, 2000; Stordal, Mykletun, & Dahl, 2003), low educational attainment (Shevlin, Rosato, Boyle, Murphy, & Boduszek, 2018), and unemployment status (Diette, Goldsmith, Hamilton, & Darity Jr, 2012) would be positively associated with membership of all non‐symptomatic classes. It was also hypothesized that childhood adversity (Suliman et al., 2009) and adult trauma exposure (McLaughlin, Conron, Koenen, & Gilman, 2010) would be positively associated all symptomatic classes.

The third aim was to test if there were significant differences in somatization and functional impairment across the resultant latent classes. Somatization was chosen as a criterion variable as it has been demonstrated to be similarly not only strongly related to depressive and anxiety disorders (Bekhuis, Boschloo, Rosmalen, & Schoevers, 2015) but also higher for comorbid anxiety and depression (Haug, Mykletun, & Dahl, 2004). It was hypothesized that all symptomatic classes would have significantly higher levels of somatization than the non‐symptomatic classes, and that the ‘comorbid’ class would have the highest levels of somatization. Functional impairment has been shown to be related to anxiety (Olatunji, Cisler, & Tolin, 2007) and depression (Greenberg, Fournier, Sisitsky, Pike, & Kessler, 2015), and so it was predicted that all the symptomatic classes would have higher levels of functional impairment.

## Method

### Participants

This study was based on a nationally representative sample of non‐institutionalized Irish adults aged 18 years and older (*N* = 1,020). Participants were recruited using an online survey company called Qualtrics, which maintains a panel of participants available for survey research that is representative of the entire adult population of Ireland. Participants were drawn from this panel using quota sampling methods to construct a sample that was representative of the general population in terms of three demographic variables: sex, age, and geographical distribution. These three sample characteristics match known population parameters as per the 2016 Irish census data. All data were collected online, and informed consent was obtained prior to a participant completing the survey. Panel members receive financial remuneration from Qualtrics for their participation in the research panel. Ethical approval for this project was provided by the Social Research Ethics Committee at Maynooth University, Ireland. The median time of completion was 22 min and Qualtrics employed checks to identify and remove any cases where participants completed the survey in a time that was deemed to be too fast to be confident that responses were trustworthy (i.e., less than 7 mins). All data were collected in February 2019.

The mean age of the sample was 43.10 years (*SD* = 15.12, range 18‐87), 51.0% were female (*n* = 520). While the age difference between men and women was statistically significant (*t*(1018) = 3.373, *p* = .01), the difference was small (mean males = 44.72 years, mean females = 41.54 years). The majority of respondents were employed full or part‐time (63.6%, *n* = 649). Over a half of participants (53.7%, *n* = 548) obtained higher education (bachelor’s degree or higher) and the majority were in a committed relationship (69.9%, *n* = 709). Full details of the sample are provided in Table 1.

**Table 1:** Sociodemographic characteristics of the sample (*N* = 1,020)

	% (*n*)
Sex	
Male	49.0 (500)
Female	51.0 (520)
Age in years	
18–24	12.3 (125)
25–34	20.2 (206)
35–44	23.5 (240)
45–54	19.1 (195)
55–64	14.1 (144)
65+	10.8 (110)
Age	*M* = 43.10, *SD* = 15.12
Region	
Dublin city and county	31.4 (320)
Leinster (not including Dublin)	22.5 (230)
Munster	26.9 (274)
Connaught	13.5 (138)
Ulster	5.7 (58)
Highest educational attainment	
Did not complete secondary school	7.1 (72)
Completed secondary school	39.2 (400)
Completed an undergraduate university degree	36.9 (376)
Completed a postgraduate university degree	16.9 (172)
Current relationship status	
In a committed relationship	69.5 (709)
Not in a committed relationship	30.5 (311)
Do you have Children?	
Yes	59.4 (606)
No	40.6 (414)
Current employment status	
Full‐time employed	45.8 (467)
Part‐time employed	17.8 (182)
Not in work (i.e., retired, student, caring for another, disabled)	27.7 (283)
Unemployed and seeking employment	8.6 (88)
Income level	
Below national mean of €45,611 per annum	71.7 (731)
Above national mean of €45,611 per annum	16.0 (163)
At or about national mean of €45,611 per annum	12.4 (126)

### Measures

Demographic variables were assessed including sex (0 = male, 1 = f emale), age (years), relationship status (in a committed relationship = 0, not in a committed relationship = 1), highest educational attainment (Completed post‐primary education = 0, Completed an undergraduate degree or higher = 1), and employment status (unemployed /seeking work/ not seeking work = 0, Full‐time/Part‐time employed = 1).

ICD‐11 Anxiety and Depression: Goldberg, Prisciandaro, and Williams (2012) and Goldberg et al., (2017) developed two 5‐item screening scales to measure ICD‐11 anxiety (Anx‐5) and depression (Dep‐5). Each scale was comprised of 2 ‘screening items’ and 3 ‘additional items’. Goldberg et al., (2012) selected these items based on an item response theory analysis of responses from 5,438 participants from primary care clinics in 14 different countries. The items were chosen from a larger pool based on the estimates of the discrimination parameter and the depression and anxiety factors were correlated highly (*r* =.88). Following this, Goldberg et al., (2017) used data from 1,488 primary care patients from four different countries, and receiver operating characteristic curves were used to test the predictive utility of the scales using diagnosis based on the Clinical Interview Schedule‐Revised (Lewis & Pelosi, 1990) and the Youden index (Youden, 1950) was used to identify the optimal cut‐off score to identify caseness. Case identification was high (89.6%) using a cut‐off score of 3 or more. For this study, the Anx‐5 and Dep‐5 items were used to develop a self‐report measure where participants were asked, ‘During the past 2 weeks, how often have you…’ and a 5‐point Likert scale was used for responses (0 = No days, 1 = Several days, 2 = Half the days, 3 = Most days, 4 = Every day). The items are presented in Table 1. The item level scores for the Anx‐5 and Dep‐5 scales were used for the main FMM analysis. The scores were also recoded into binary variables to indicate endorsement to calculate rates of probable anxiety and depression. The ICD‐11 description describes symptoms as having to occur ‘more days than not’, and so item scores of 3 (Most days) or 4 (Every day) were considered to reflect endorsement and were coded as ‘1’, and lower scores (0 = No days, 1 = Several days, 2 = Half the days) were coded as ‘0’. In order to categorize participants as meeting the criteria for anxiety or depression, three or more symptoms needed to be endorsed (Goldberg et al., 2017), including at least one of the ‘screening items’. This maps onto the ICD‐11 description for depression (6A70) that requires ‘depressed mood or diminished interest in activities’ and ‘…accompanied by other symptoms’. Similarly, the ICD‐11 description for generalized anxiety disorder (6B00) requires ‘general apprehension (i.e., “free‐floating anxiety”) or excessive worry’ that occur ‘…together with additional symptoms’.

Childhood adversity: The Adverse Childhood Experiences scale (ACE; Felitti et al., 1998) is a 10‐item self‐report measure of childhood adversity that measures occurrences of emotional, physical abuse, sexual abuse, physical neglect, and household dysfunction. Responses were binary scored (Yes = 1, No = 0) and summed, with a possible range of scores of 0 to 10. Previous research has provided evidence of the validity of ACE scores (Kazeem, 2015).

Adult Traumatic Exposure: The International Trauma Exposure Measure (ITEM: Hyland et al., 2021) is a checklist of 21 traumatic life events, 16 of which measure ‘Criterion A’ traumatic exposure, similar to Life Event Checklist (Weathers et al., 2013), and five items measured traumatic experiences that pose threats to psychological safety such as repeatedly bullied, neglected, ignored, rejected, or isolated, and stalked. Participants were asked to indicate experience of each of these events when ‘older than 18 years of age’ and responses were binary scored (Yes = 1, No = 0) and summed, with a possible range of scores of 0 to 21.

Work and Social Adjustment symptoms: The Work and Social Adjustment Scale (WSAS; Mundt, Marks, Shear, & Greist, 2002) is a 5‐item self‐report scale that examines one’s perceived functional impairment in the areas of work, home management, social leisure activities, private leisure activities, and relationships with others. Each item of the scale is associated with one of the areas. Participants are asked ‘…determine on the scale provided how much your problem affects your ability to carry out the activity’. Responses range from ‘Not at all’ (1) to ‘Very severely’ (7) with higher scores reflecting higher levels of functional impairment. Cronbach’s α in the present sample was excellent (α = .92).

Somatization: The Trauma Symptom Inventory (TSI‐2: Briere, 2011) is a 136‐item self‐report scale measuring 12 different sets of symptoms that can occur following traumatic exposure. In this study, we selected the 10‐item Somatic Preoccupation scale. Participants were provided with the following instructions, ‘Below is a list of problems and complaints that people sometimes have. Please read each problem carefully. Then, indicate how often each of the following experiences have happened to you in the last six months’ and are presented with a list of physical problems: (1). Aches or pains, (2). Lower back pain, (3). Muscle spasms, (4). Chest pain, (5). Dizziness, (6). Nausea or an upset stomach, (7). Indigestion, (8). Ringing in your ears, (9). Difficulty swallowing, and (10). Trouble keeping your balance. The items are rated on a 4‐point Likert scale (0‐3) ranging from ‘Never’ to ‘Often’, and item‐level scores were used in the analysis. The reliability of this sample was high, with a Cronbach’s α of .87.

### Analysis

The main analyses were conducted in three linked phases using Mplus 8.1 (Muthén & Muthén, 2017). First, following the guidelines proposed by Clark et al., (2013), the initial analyses involved fitting confirmatory factor analysis (CFA) and latent profile analysis (LPA) models to the data from the Anx‐5 and Dep‐5 items. CFA was performed to establish the appropriate latent structure of the items and two models were tested: a one‐factor model with all items loading onto one factor, and a correlated two‐factor model with the five depression items loading on a ‘depression’ factor, and the five anxiety items loading onto an ‘anxiety’ factor. All unique variances were uncorrelated. Given the limited amount of psychometric research on the performance of the Anx‐5 and Dep‐5 items we also tested a series of exploratory factor analysis (EFA) models on order to determine if there were other plausible models that could be added to those being tested in the CFA phase. Models with one through to three factors were tested, with Geomin rotation, to examine the plausibility of a non‐simple structure two‐factor model or a model with more than two factors variables. The fit of the EFA and CFA models were assessed using standard criteria: a non‐significant chi‐square (χ^2^) test, Comparative Fit Index (CFI; Bentler, 1990), and Tucker Lewis Index (TLI; Tucker & Lewis, 1973) values greater than.90; Root‐Mean‐Square Error of Approximation (RMSEA: Steiger, 1990) with 90% confidence intervals (RMSEA 90% CI); and Standardized Root‐Mean‐Square Residual (SRMR) values of .08 or less reflect acceptable model fit (Hu & Bentler, 1999). The models were also compared using three information theory based fit statistics with lower values indicating better fit: the Akaike Information Criterion (AIC; Akaike, 1987), the Bayesian Information Criterion (BIC; Schwartz, 1978), and the sample size adjusted Bayesian Information Criterion (ssaBIC; Sclove, 1987).

For the latent profile analysis, models with 2 through to 6 classes were fitted. This was done to determine if there was significant heterogeneity in the responses. Within‐class correlations were all fixed to zero. Model fit was assessed using the information theory based fit statistics and the Lo‐Mendell‐Rubin adjusted likelihood ratio test (LMR‐A; Lo, Mendell, & Rubin, 2001). The LMR‐A was used to compare models with increasing numbers of classes, and when a non‐significant value (*p *> .05) occurs, this suggests that the model with one less class should be accepted. For LPA models it is common that the information theory based fit statistics do not reach a minimum among the models tested and so a ‘…diminishing gains in model fit’ (Masyn, 2013, p. 572) approach has been advocated where the ‘best’ model is indicated when the decrease in values becomes relatively small, and the LRT‐A can also be used in the decision making process (see Marsh, Lüdtke, Trautwein, & Morin, 2009). The entropy for each solution reflects the degree of correct classification of participants with values closer to one being indicative of better classification (Ramaswamy, DeSarbo, Reibstein, & Robinson, 1993). The EFA, CFA, and LPA models were estimated using robust maximum likelihood (Yuan & Bentler, 2000). To avoid LPA solutions based on local maxima, 2000 random sets of starting values were initially used and 100 final stage optimizations.

When the best fitting CFA model was determined, and if heterogeneity was evident based on the LPA, FMMs were deployed testing increasing number of classes (2 to 7). Type‐2 FMMs (Clark et al., 2013) were used with class invariant intercepts, class invariant factor loadings, class invariant factor covariance matrices, and class specific factor means being estimated. The factor means for a reference class were fixed at zero. This specification was used as it maximized statistical power. An alternative specification (e.g., FMM‐3) would require the estimation of all intercepts in each class, whereas FMM‐2 only estimates factor means. Fewer free parameters are, therefore, being estimated when using FMM‐2. The FMM‐2 model is based on the idea that skewed distributions may reflect a ‘mixture’ of separate normal distributions (representing different homogeneous classes), and these vary along the factor distributions. However, it is also the case that classes may just represent ‘cut‐points’ along a single homogeneous non‐normal distribution, so as recommended by Bauer and Curran (2003), the associations between the resultant classes and theoretically related predictor variables were assessed to determine if these were significant and if there was evidence of specificity. The estimation and assessment of model fit for the FMMs was the same as for the LPAs.

In the second phase, the covariates (sex, age, relationship status, education, employment, adult trauma, adverse childhood experiences) were used as predictors of the latent classes, analogous to a multinomial logistic regression, using the R3STEP method (Asparouhov & Muthén, 2014). This approach accounts for the uncertainty of class membership and does not influence the estimation of the latent class part of the model (Asparouhov & Muthén, 2014).

In the third phase, the sum scores of WSAS and TSI‐2 somatization items were specified as distal outcome being predicted by the latent classes using the DU3STEP method which assumes unequal means and variances across classes. This tested for the equality of means of the somatization items across the levels of the FMM classes. A Wald test is used for the overall test of equal means and is supplemented with pairwise comparisons (Asparouhov & Muthén, 2014).

## Results

The mean scores and endorsement rates for the Anx‐5 and Dep‐5 items are reported in Table 2. The mean scores for all items were low, mostly below 1 indicating that the symptoms were being reported to have occurred on average ‘No days’ in the previous two weeks. The ‘Feeling nervous or anxious’ item had the highest mean score of 1.03, indicating that this was experienced, on average, ‘Several days’ during the previous 2 weeks. The skew statistics showed that all the univariate distributions were positively skewed, and the degree of skew (> 1) was indicative of significant departure from normality.

**Table 2:** Mean scores and endorsement rates for ICD‐11 anxiety and depression scales

During the past 2 weeks, how often have you…	Mean (*SD*)	Endorsement (%)	Skew
Anxiety			
Anx 1: Felt nervous or anxious?	1.03 (1.21)	14.9	1.15
Anx 2: Been unable to control your worrying?	.89 (1.22)	14.0	1.33
Anx 3: Had trouble relaxing?	.97 (1.19)	13.9	1.24
Anx 4: Felt so restless that it was hard to keep still?	.65 (1.03)	8.5	1.73
Anx 5: Felt afraid that something awful might happen?	.75 (1.16)	11.8	1.54
Total summed score	4.30 (5.16)		
Depression			
Dep 1: Been feeling down or depressed?	.95 (1.14)	13.2	1.20
Dep 2: Experienced less interest or pleasure from normal activities?	.94 (1.14)	12.6	1.22
Dep 3: Experienced problems with your concentration?	.91 (1.17)	13.4	1.28
Dep 4: Experienced feelings of worthlessness?	.80 (1.18)	12.3	1.45
Dep 5: Felt that you wanted to die or had thoughts of death?	.46 (.98)	7.1	2.31
Total summed score	4.06 (4.91)		

The endorsement rates (‘Most days’ or ‘Every day’) were generally higher for the anxiety symptoms than the depression symptoms. The rate of probable anxiety was 11.3%, and 10.3% for depression. There were significant gender differences with more females meeting the criteria for depression (females = 13.7%, males = 6.8%; *χ^2^* (1) = 12.97, *p* < .001) and anxiety (females = 16.2%, males = 6.2%; *χ^2^* (1) = 25.25, *p *< .001). There was a high level of co‐occurring anxiety and depression (*χ^2^* (1) = 384.14, *p* < .001) with 7.1% meeting the criteria for both disorders while 4.2% and 3.2% were ‘unique’ cases of anxiety and depression, respectively.

The number of reported adverse childhood experiences, as measured by the ACE scale, ranged from 0 to 10 and the mean was 2.08 (*SD* = 2.31, *Mdn* = 1.00). Approximately one‐third (34.8%) reported no adverse childhood experiences, 17.8% reported one event, 13.6% reported two events, 9.7% reported three events, and 24.0% reported four or more events. The number of reported adult traumatic experiences, as measured by the ITEM, ranged from 0 to 13 and the mean was 2.32 (*SD* = 2.42, *Mdn* = 2.00). Less than one‐third (29.8%) reported no traumatic experiences, 17.6% reported one trauma, 14.9% reported two, 10.8% reported three, and 27.0% reported four or more. The scores for the TSI‐2 somatization scale ranged from 0 to 29 and the mean was 9.93 (*SD* = 6.52). WSAS scores ranged from 5 to 35 and the mean was 23.19 (*SD* = 8.82).

The EFA fit statistics for the one‐factor (*χ^2^* (35) = 260.098, *p *< .001; CFI = .945, TLI = .929; RMSEA = .079 (90%CI = .071, .089), SRMR = .030) two‐factor (*χ^2^* (26) = 161.138, *p* < .001; CFI = .967, TLI = .943; RMSEA = .071 (90%CI = .061, .082), SRMR = .019) and three‐factor model (*χ^2^* (18) = 69.821, *p *< .001; CFI = .987, TLI = .968; RMSEA = .053 (90%CI = .040, .067), SRMR = .012) all indicated reasonable fit. Chi‐square difference tests indicated that the two‐factor was better than the one‐factor model (Δ*χ ^2^* = 79.612 (Δdf = 9), *p* < .001) and the three‐factor model was better than the two‐factor model (Δ*χ ^2^* = 79.612 (Δdf = 9), *p *< .001). The two‐factor model solution was clearly defined with the 5 depression items loading on the Depression factor with loadings ranging from .697 (*p *< .05) to .949 (*p *< .05) and the 5 anxiety items loading on the Anxiety factor with loadings ranging from .641 (*p* < .05) to .930 (*p* < .05) and there was only one significant cross loading with Dep 2 (Experienced less interest or pleasure from normal activities) loading on the Anxiety factor at .393 (*p* < .05). The three‐factor solution was poorly defined with only two significant loadings on the third factor (Dep 4 = .300, *p* < .05 & Dep 5 = .390, *p *< .05), and this factor did not correlate significantly with the first two factors that remained clearly defined. On the basis of this no models were carried forward to the CFA phase of analysis.

The fit statistics for the one‐factor (*χ^2^* (35) = 260.098, *p *< .001; CFI = .945, TLI = .929; RMSEA = .079 (90%CI = .071,.089), SRMR = .030) and two‐factor (*χ^2^* (34) = 151.114, *p *< .001; CFI = .971, TLI = .962; RMSEA = .058 (90%CI = .049,.068), SRMR = .024) CFA models indicted acceptable fit for both. However, the BIC was lower for the two‐factor model and it was therefore judged to be the best model. The standardized factor loadings for the depression items were all positive and ranged from.71 to.90 and all were statistically significant (*p *< .001). The standardized factor loadings for the anxiety items were all positive and ranged from.76 to.90 and all were statistically significant (*p *< .001). The correlation between the anxiety and depression latent variables was very strong (*r* = .93, *p *< .001). The fit statistics for the LPA models suggested that the four‐class model had optimal fit. While the BIC continued to decrease with additional classes, a non‐significant LMR‐A result was obtained for the five‐class solution, indicating that the four‐class solution was superior. The CFA solution indicated that the depression and anxiety items measure two separate‐but‐highly correlated dimensions, and the LPA solution showed that there was significant heterogeneity in responses. These findings supported the use of an FMM approach.

**Figure 1:**
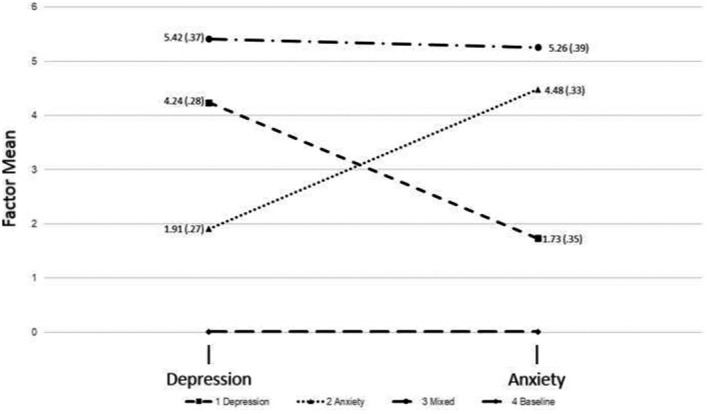
Profile plot of latent variable means (Standard Errors) from 4‐class factor mixture model.

The fit indices (Table 3) for all of the two‐factor FMMs had lower BIC values compared to the CFA models and the corresponding LPA models. This indicated that the FMM solutions were superior to the CFA and LPA solutions. The AIC, BIC, and ssaBIC values decreased for models with two through seven classes, however, the decrease was markedly smaller after four classes. The decrease in the BIC from the three to four class solutions (ΔBIC = 225.242) was much larger than the decrease from the four to five class solution (ΔBIC = 67.909), and the LMR‐A became statistically significant at the five‐class model. The five‐class solution also produced out of range estimates. The entropy value (.93) for four‐class models was high. Thus, the two‐factor four‐class model was deemed to be the best balance between model complexity and relative model fit (see Figure 1).

**Table 3:** Fit statistics for the CFA, LCA, and FMM of ICD‐11 anxiety and depression items

Model	Log‐likelihood	AIC	BIC	ssaBIC	Entropy	LMR‐A (*p*)
CFA						
1 factor	−11310.729	22681.458	22829.285	22734.002	–	–
2 factors	−11184.370	22430.740	22583.494	22485.035	–	–
LPA						
2 classes	−12362.666	24787.332	24940.086	24841.627	.987	.000
3 classes	−11496.482	23076.964	23283.921	23150.525	.946	.001
4 classes	−11026.380	22158.761	22419.921	22251.588	.937	.009
5 classes	−10802.090	21732.180	22047.544	21844.274	.943	.733
6 classes						
FMM						
2 factors 2 classes	−10950.024	21968.047	22135.584	22027.597	.931	.000
2 factors 3 classes	−10863.474	21800.948	21983.267	21865.752	.923	.071
2 factors 4 classes	−10740.461	21560.923	21758.025	21630.981	.938	.005
2 factors 5 classes	−10696.115	21478.231	21690.116	21553.544	.932	.150
2 factors 6 classes	−10658.671	21409.342	21636.010	21489.910	.934	.054
2 factors 7 classes	−10647.956	21393.912	21635.363	21479.734	.933	.012

Note

AIC = Akaike Information Criterion; BIC = Bayesian Information Criterion; ssaBIC = sample size adjusted Bayesian Information Criterion, LMR‐A Lo‐Mendell‐Rubin adjusted likelihood ratio test. Best‐fitting models for each approach (CFA, LPA, FMM) shown in bold.

Class 1 (*n* = 65, 6.4%) was characterized by a low factor mean for anxiety (*M* = 1.73, SE = .35) and a high factor mean for depression (*M* = 4.24, SE = .28), and was thus labelled the ‘Depression’ class. Class 2 (*n* = 64, 6.3%) was characterized by a high factor mean for anxiety (*M* = 4.48, SE = .33) and a low factor mean for depression (*M* = 1.91, SE = .27), and was thus labelled the ‘Anxiety’ class. Class 3 (*n* = 120, 11.7%) was characterized high factor means for both anxiety (*M* = 5.26, SE = .39) and depression (*M* = 5.42, SE = .37) and was labelled as the ‘comorbid’ class. Class 4 (*n* = 771, 75.6%) was the largest, and was the reference class with factor means fixed at zero. This was labelled the ‘Non‐symptomatic’ class.

The predictors (sex, age, relationship status, education, employment, adult trauma, adverse childhood experiences) were added to the model, analogous to a multinomial logistic regression, with class membership as the dependant variable and the baseline class as the reference class. The results are presented as adjusted odds ratios (OR) in Table 4. Being female increased the odds of membership in the comorbid class (OR = 1.92) and the anxiety class (OR = 2.48) compared to the non‐symptomatic class. Increased age reduced the odds of being in the depression (OR = 0.95), anxiety (OR = 0.95) and comorbid class (OR = 0.95). Educational attainment was only associated with the depression class with higher educational attainment associated with lower odds of class membership (OR = 0.51). Childhood adversity and adult trauma increased the odds of being in all classes, compared to the non‐symptomatic class, with odds ratios ranging from 1.16 to 1.51.

**Table 4:** Demographic and trauma‐related predictors of class membership

Predictor	Class 1: Depression OR (95% CI)	Class 2: Anxiety OR (95% CI)	Class 3: Comorbid OR (95% CI)
Gender (female)	1.29 (.67, 2.47)	2.48 (1.28, 4.79)**	1.92 (1.12, 3.29)**
Age	.95 (.93, .97) **	.95 (.93, .97)**	.95 (.93, .97)**
Relationship Status (not in a committed relationship)	1.84 (1.00, 3.39)	1.34 (.70, 2.58)	1.26 (.72, 2.19)
Education (BSc or higher)	.51 (.27, .96) *	.90 (.48, 1.68)	.97 (0.58, 1.6)
Employment (being employed part‐ or full time)	.55 (.29, 1.05)	.98 (.51, 1.86)	.73 (0.43, 1.23)
Total Adult trauma	1.16 (1.02, 1.31) *	1.17 (1.04, 1.31)**	1.18 (1.07, 1.29)**
Total ACE	1.22 (1.06, 1.4) **	1.31 (1.17, 1.46)**	1.51 (1.36, 1.68)**

Note

Class 4 (Baseline) is the reference category; *, significant at *p *< .05; **, significant at *p *< .01.

The sum scores of Work and Social Adjustment Scale and Somatization scale (TSI‐2) were added as distal outcomes, and descriptive statistics and results from the tests of mean differences across classes are presented in Table 5. There was a significant main effect for both scales. For WSAS, the mean scores for the comorbid class and the depression class were significantly higher than both the anxiety and non‐symptomatic classes, with no significant difference between the comorbid class and the depression class. In the case of somatization, the comorbid class presented the highest mean with significant differences compared to the rest of the classes. There were no significant differences between the depression and anxiety classes but both were significantly higher than the non‐symptomatic class.

**Table 5:** Means (standard error) and tests of differences on functioning and somatization items across latent classes

	Class 1: Depression		Class 2: Anxiety		Class 3: Comorbid		Class 4: Non‐symptomatic		Overall Wald test	Pairwise comparison (*p *< .05)
	Mean	(*SE*)	Mean	( *SE* )	Mean	(*SE*)	Mean	(*SE*)		
Functional Impairment (WSAS)	26.082	.705	22.751	1.296	26.373	.580	14.099	.298	558.309 *p *< .001	1,3 > 2>4
Somatization (TSI‐2)	12.895	.916	13.848	.870	17.837	.595	8.112	.213	277.335 *p *< .001	3 > 1,2 > 4

As a follow‐up analysis, a bi‐factor model was also fitted to further examine the latent structure of the anxiety and depression items. This model specified an Anxiety latent variable measured by the Anx‐5 items and a Depression latent variable measured by the Dep‐5 items, and a ‘General’ latent variable on which all the items loaded. The latent variables were specified to be uncorrelated (Reise, 2012) and all unique variances were uncorrelated. The fit of this model was acceptable (*χ^2^* (25) = 133.210, *p *< .001; CFI = .973, TLI = .952; RMSEA = .065 (90%CI = 055, .076), SRMR = .020: BIC = 22564.091). The standardized factor loadings on the General factor ranged from.711 to.900 and were all significant (*p* < .001), and from.254 to.369 for the Anxiety factor (all *p* < .001). Only two loadings, Dep 1 (.284, *p* < .001) and Dep2 (.319, *p* < .001), were significant for the Depression factor. The differences in the loadings were reflected in the Explained Common Variance (Stucky & Edelen, 2014) estimates, the proportion of all common variance explained by the factors, as it was high for the General factor (.907) and lower for the Anxiety (.068) and Depression factors (.025). Overall, this means that the General factor accounted for the majority of the common variance and the other factors were relatively unimportant; this does not support the multi‐dimensional nature of the data.

## Discussion

The primary aim of this study was to model endorsement of ICD‐11 anxiety and depression symptoms, using FMM, with data from a representative sample of the Irish adult population to determine if there was a profile consistent with ICD‐11 description of ‘Mixed depressive and anxiety disorder’ (MDAD). Contrary to the ICD‐11 description which implies the presence of symptoms of anxiety and depression at a lower than clinically significant level, but at a level high enough to warrant treatment, the results of the FMM suggested that there is no homogeneous group with sub‐clinical levels of endorsement for both anxiety and depression. Indeed, the results indicated that that the comorbid class was the largest symptomatic class, and the ‘pure’ anxiety and depression classes were smaller. Whereas the ICD‐11 proposes MDAD as a special case of anxiety and depression, these results suggest the reverse; that anxiety and depression are special cases of the more general disorder MDAD.

The second aim of the study was to assess the relationship between demographic and stress/trauma‐related variables and the classes obtained from the FMM. There was a negative association between age and membership of the depression, anxiety, and comorbid classes which is consistent with the extant research on anxiety and depression. Being female was significantly associated with membership of the anxiety and comorbid classes. The effect was not found for the ‘depression’ class which contradicts previous findings (Albert, 2015; Seedat et al., 2009). Interestingly, no effects were found for employment nor an individual’s relationship status; these were previously shown to be protective against poor psychological well‐being (Diette et al., 2012). However, many previous studies finding protective effects for higher education, being employed and in a committed relationship did not control for childhood and adult traumatic events (e.g., Boyle, Murphy, Rosato, Boduszek, & Shevlin, 2018; Johansson, Carlbring, Heedman, Paxling, & Andersson, 2013; Pillay & Sargent, 1999). Traumatic events experienced in childhood and adulthood both had significant positive effects when predicting belonging to each of the symptomatic classes. Both cumulative trauma and singular traumatic events have been previously shown to predict anxiety and depression (Lipsky, Kernic, Qiu, & Hasin, 2016; Slopen, Fitzmaurice, Williams, & Gilman, 2012; Suliman et al., 2009). Overall, the associations between the demographic and stress/trauma‐related variables were consistent with the extant research literature on anxiety and depression, and therefore provides some evidence of the validity of the FMM solution.

It was predicted that symptomatic classes would have higher levels of somatization and functional impairment, and this was supported. Table 4 shows that the non‐symptomatic class had significantly lower somatization and functional impairment scores than all other classes. The anxiety class is associated with lower somatization and functional impairment than the comorbid class, although there was no difference between the depression and comorbid class in terms of functional impairment. These findings are consistent with those reported by de Waal, Arnold, Eekhof, and van Hemert (2004), who reported significant co‐occurrence of somatic problems and anxiety and depression, with a proportional increase in functional impairment, in a sample of general practitioner patients. The results of this study suggest that the co‐occurrence of anxiety and depression is associated with additional physical and functioning burden compared to anxiety and depression separately, consistent with the findings of Löwe et al., (2008).

An interesting finding was that the 1 through 3 factor EFA models all fitted the data well, as did the 1 and 2 factor CFA models. The bi‐factor model was also a well‐fitting model and this could be considered a hybrid of the first‐order unidimensional model (represented by the General factor) and multidimensional models (with Anxiety and Depression as specific factors). This is analogous to the many studies that reported different well‐fitting factor analytic models of posttraumatic stress disorder symptoms (see Elhai & Palmieri, 2011), and Shevlin and Elklit (2012) proposed that there may not be one ‘correct’ model, but rather that the models represent different sub‐populations. They showed that different symptom structures were associated with different groups based on the type of trauma exposure. On the basis of this, it could be that the bi‐factor model fits the data well because it represents different sub‐populations: the General factor captures variation in all anxiety and depression symptoms and so could represent the baseline and comorbid classes (differing only in severity), and the specific factors represent the ‘anxiety only’ and ‘depression only’ classes. This possible explanation is given support by Raykov, Marcoulides, Menold, and Harrison (2019) who showed that population heterogeneity can make bi‐factor solutions ‘spuriously plausible’ (p. 110).

The findings of this study should be interpreted with some limitations in mind. First, the ICD‐11 Anx‐5 and Dep‐5 scales have not been extensively validated and replication is required with other measures of depression and anxiety. Second, somatization was specified as an outcome of anxiety and depression but the temporal ordering among these variables could not be established. It needs to be acknowledged that there is an ongoing debate on the causal relationship between psychological disorders and their somatic counterparts, however their co‐occurrence is well described (Michaelides & Zis, 2019). Additionally, somatization was the only psychological outcome variable used in this study, it would be interesting for future research to investigate the distress and functional impairment that may be associated with comorbid anxiety/depression, and if it differs from pure anxiety or pure depression (Rivas‐Vazquez, Saffa‐Biller, Ruiz, Blais, & Rivas‐Vazquez, 2004). Finally, there is evidence that mixture models can give rise to spurious classes (Bauer & Curran, 2003; Muthén & Asparouhov, 2015) where the classes represent groups that differ only in terms of severity rather than ‘types’ that represent different sub‐populations. However, the different classes that were reported in this study differed both qualitatively and quantitatively, and there was evidence of differential associations with the predictor variables, which supports the validity of the solution (Horn, 2000).

In conclusion, this study failed to identify a sub‐population with a sub‐clinical symptom profile consistent with ICD‐11’s ‘Mixed depressive and anxiety disorder’; rather the ‘Comorbid’ class was the largest of the symptomatic classes and was characterized by levels of both anxiety and depression that were higher than the classes that indicative of ‘pure’ anxiety and depression. This ‘Comorbid’ class was associated with being female, younger, and having experienced trauma in childhood and adulthood; it was also associated with significantly higher levels of somatic problems. The implication is that co‐occurring anxiety and depression may be more common than either disorder individually, and that this comorbidity carries additional psychological and health consequences. Indeed, if these results were to be replicated consistently it would suggest that comorbid anxiety and depression could be a primary diagnosis, of which pure anxiety and depression would be special cases.

## Conflict of interest

All authors declare no conflict of interest.

## Data availability statement

The data that support the findings of this study are available from the corresponding author upon reasonable request.

## Author contributions

Marcin Owczarek (Investigation; Writing – original draft; Writing – review & editing) Menachem Ben‐Ezra (Data curation; Funding acquisition; Writing – review & editing) Thanos Karatzias (Data curation; Funding acquisition; Investigation; Writing – review & editing) Mark Shevlin (Conceptualization; Formal analysis; Methodology; Supervision; Writing – original draft) Philip Hyland (Conceptualization; Formal analysis; Writing – original draft; Writing – review & editing) Emma Nolan (Project administration; Writing – original draft; Writing – review & editing).
